# Supplementary material for: DNA methylation repels targeting of Arabidopsis REF6
Source: Nat Commun. 2019 May 2;10:2063. doi: 10.1038/s41467-019-10026-1 (PMC6497721; doi:10.1038/s41467-019-10026-1)
Supplement: Supplementary file 1 — Supplementary Information [file 41467_2019_10026_MOESM1_ESM.pdf]

Supplementary Information

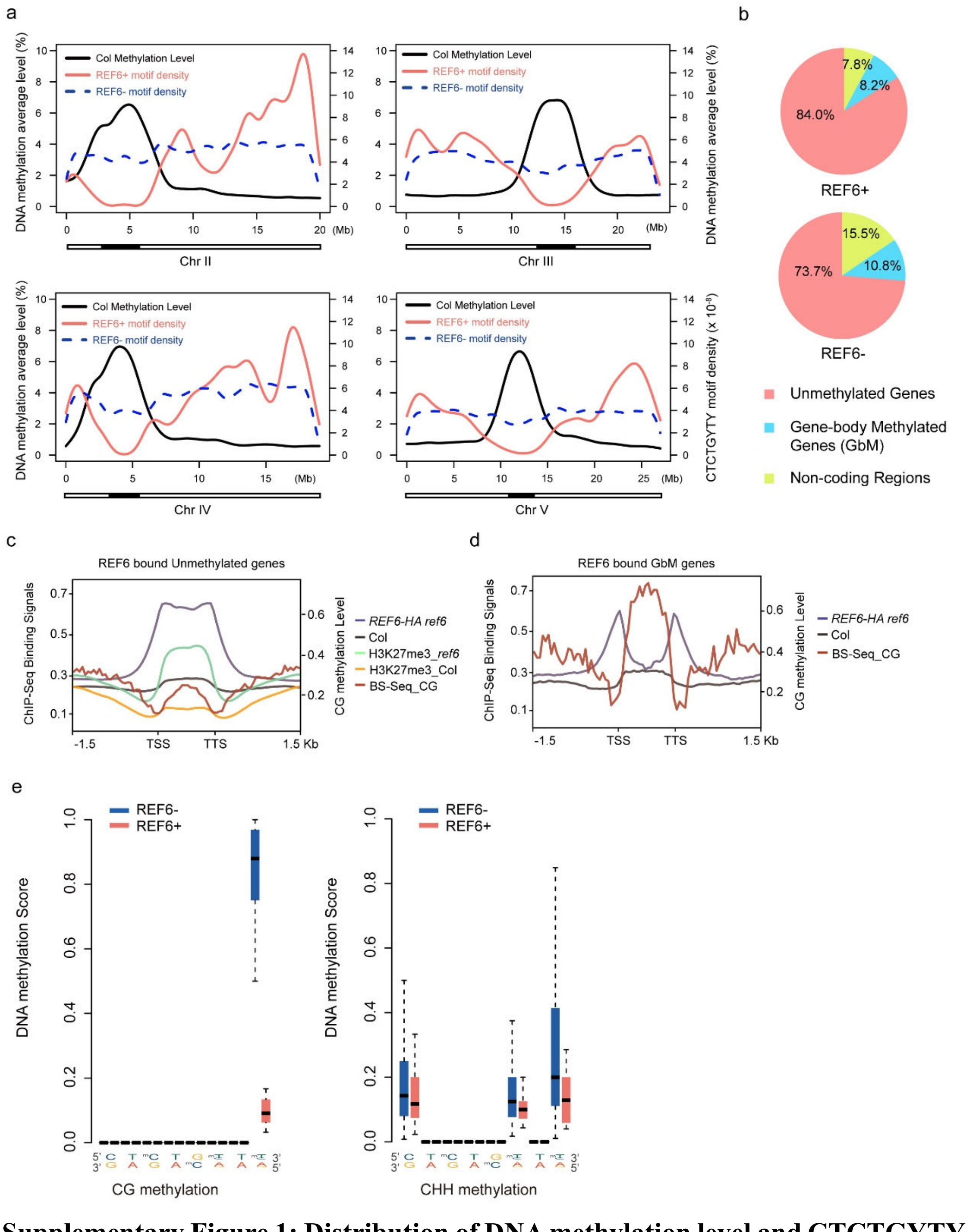

Supplementary Figure 1: Distribution of DNA methylation level and CTCTGYTY motifs across the *Arabidopsis* chromosome.

(a) DNA methylation level, as well as REF6-bound and -unbound motif density in Chromosomes II to V. Black bars below the graphs show the positions of heterochromatin.

(**b**) Pie diagram showing categories of targeted genes associated with REF6-bound and -unbound regions.

(**c,d**) Average profiles of REF6 ChIP-seq, H3K27me3 ChIP-seq, and DNA bisulfite sequencing signal at unmethylated genes (**c**) and gene-body methylated (GbM) genes (**d**) around the transcription start site (TSS) and transcription termination site (TTS).

(e) DNA methylation levels at CTCTGYTY motifs in REF6-bound (REF6+) and unbound (REF6-) regions. Box plots display the median (center line) and interquartile range (IQR; from the 25th to 75th percentile), and the whiskers represent the minimum and maximum of DNA methylation score from 0 to 1.

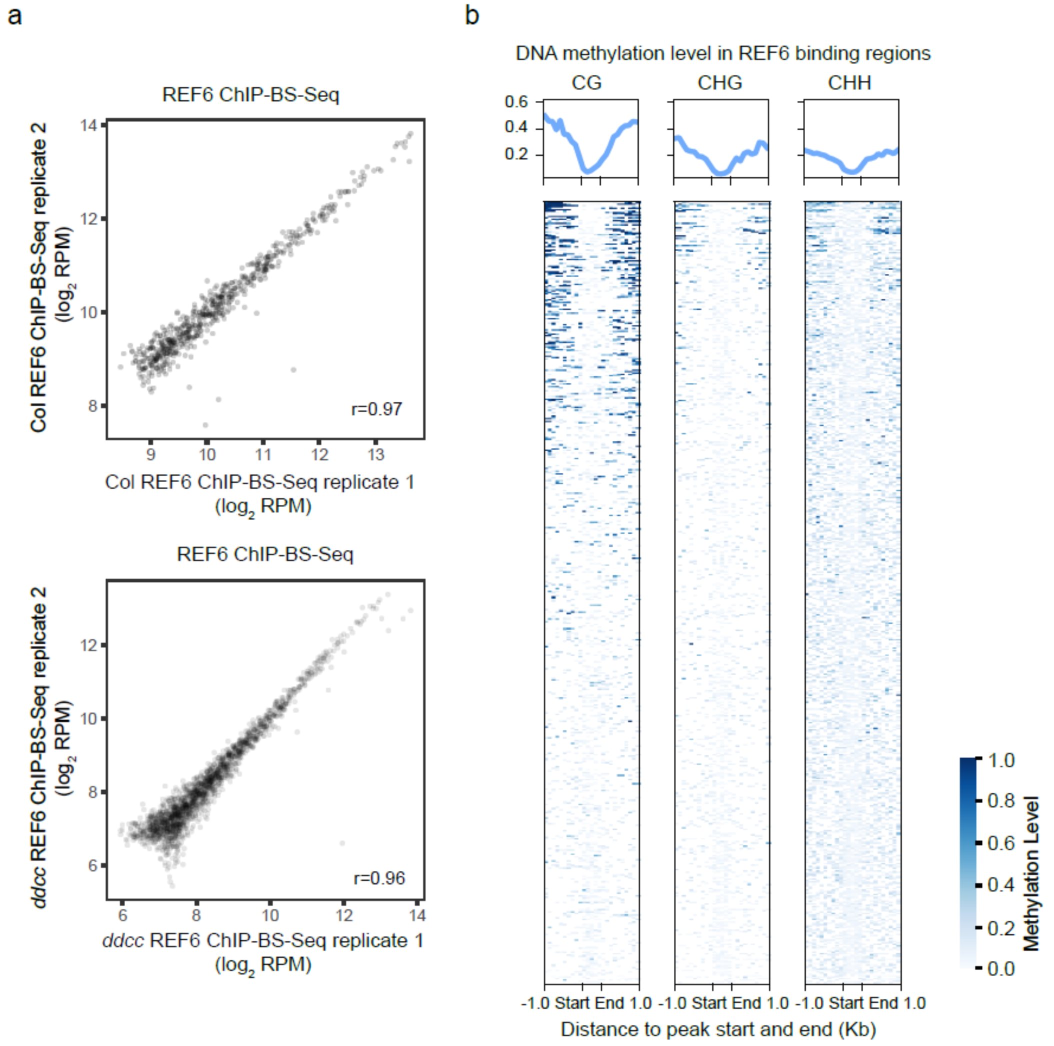

Supplementary Figure 2. ChIP-BS-seq shows REF6 prefer to bind hypomethylated regions in *Arabidopsis* genome.

(a) Scatterplots of normalized ChIP-BS-seq signal intensity in log_2 scale over merged peak regions of two replicates showing the ChIP signal in different genotype has a high correlation in replicates.

(b) Heat maps of DNA methylation levels in 1.0 Kb surrounding REF6 binding peaks.

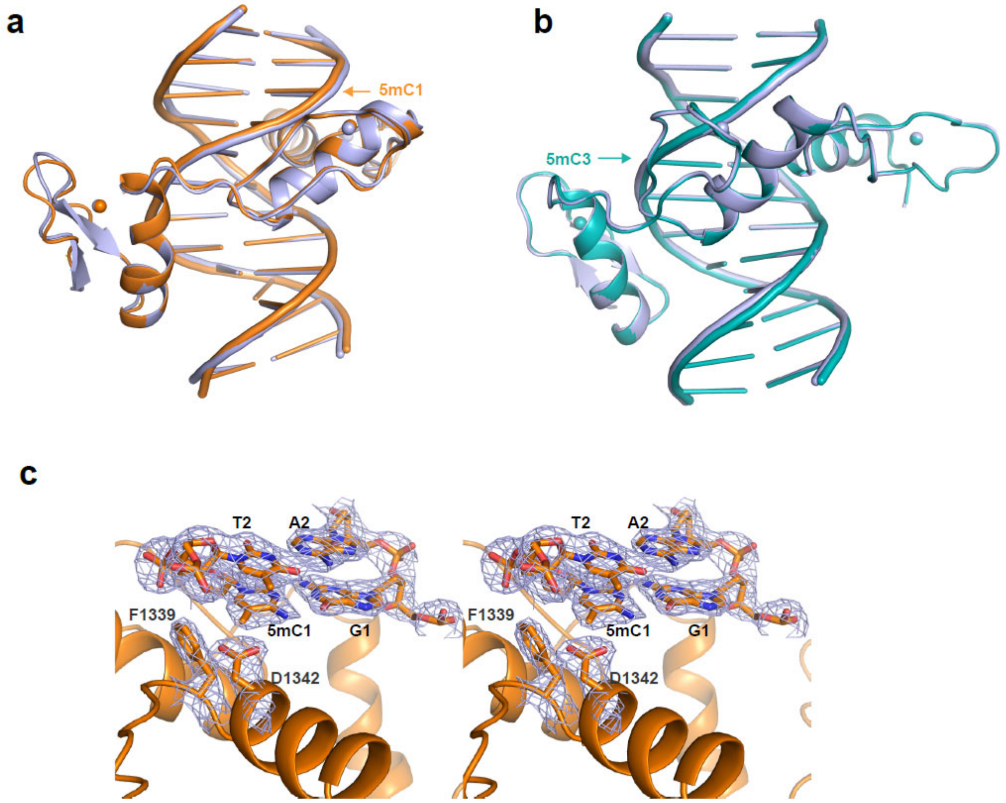

Supplementary Figure 3. Cytosine methylation influences REF6 binding.

(a) Structural superimposition of ZF2-4-*NAC004*_5mC$_1$ (orange) and ZnF2-4-*NAC004* (light blue).

(b) Structural superimposition of ZF2-4-*NAC004*_5mC$_3$ (teal) and ZnF2-4-*NAC004* (light blue).

(c) Stereoview showing the detailed interaction with the methy-group in the ZF2-4-NAC004_5mC$_1$ complex. ZF2-4 is shown as cartoon in orange. DNAs are shown as sticks. The 2Fo-Fc electron density maps are colored in blue and contoured at 1.5 sigma level.

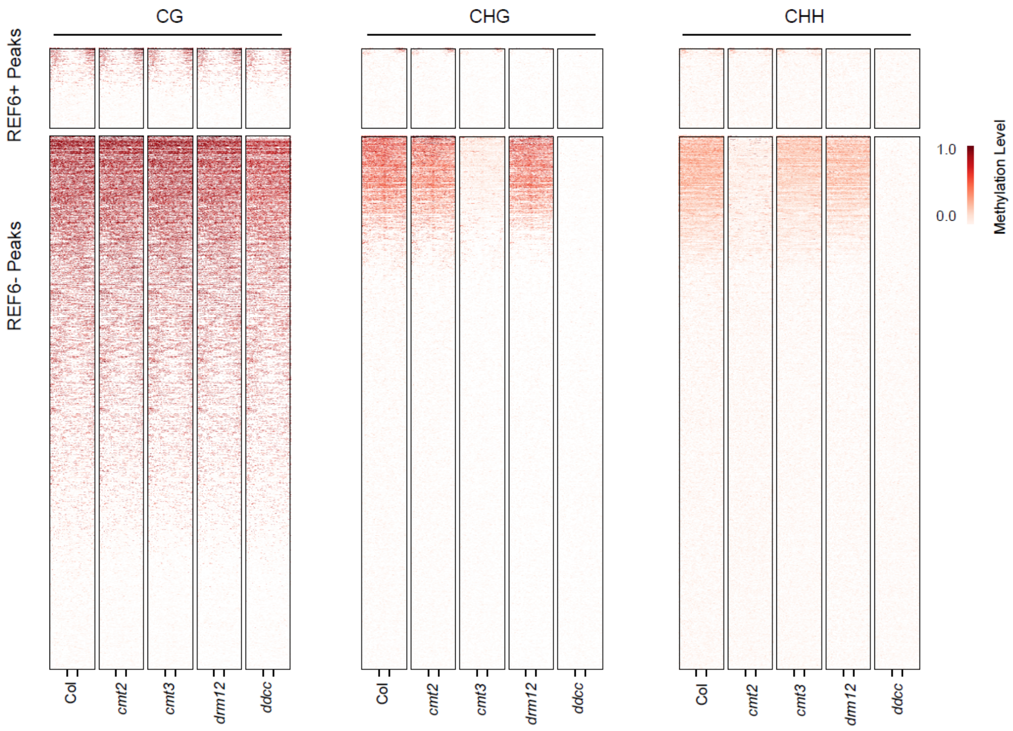

Supplementary Figure 4. Heat maps of DNA methylation levels around REF6-bound (REF6+) and -unbound (REF6-) regions in different mutants.

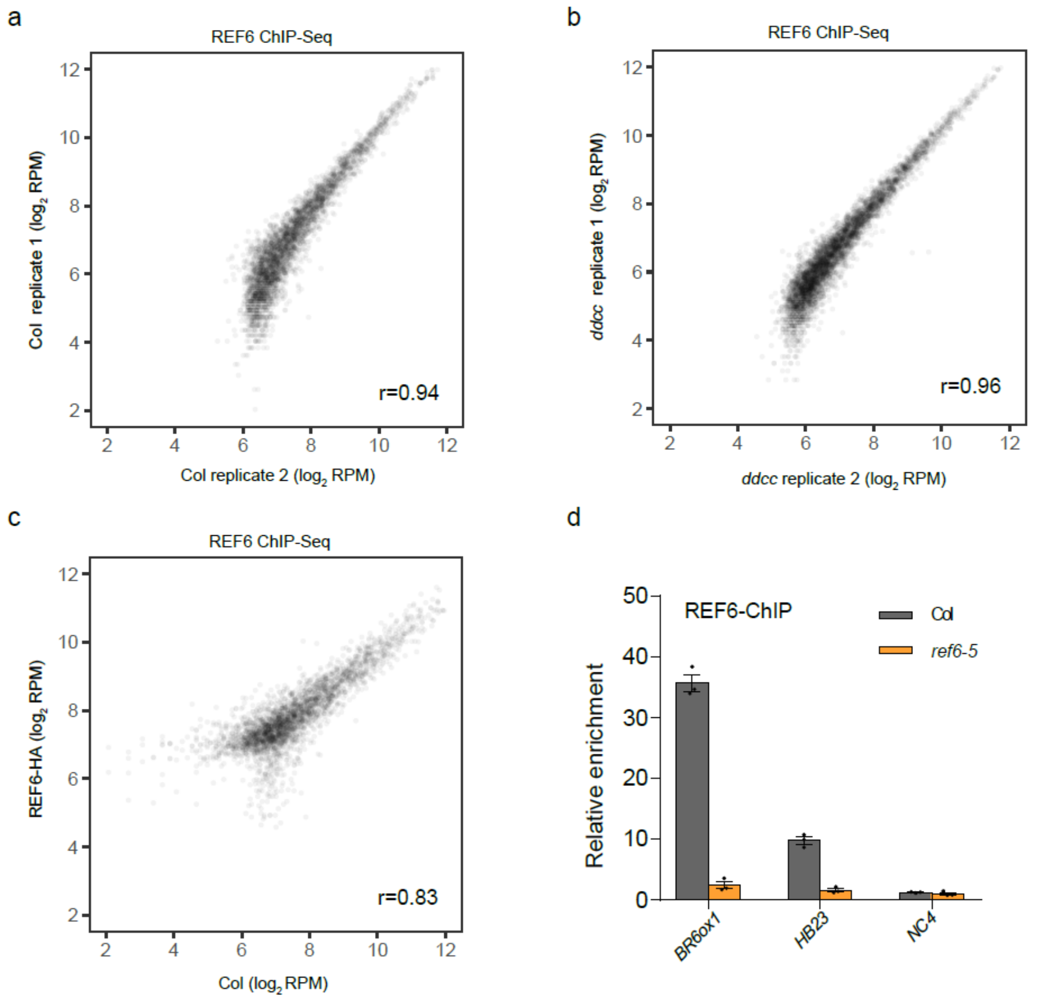

Supplementary Figure 5. ChIP-seq with anti-REF6 antibody efficiently enriches REF6 target genes.

Scatterplots of normalized ChIP-seq signal intensity in log_2 scale over all regions between two Col replicates **(a)**, two *ddcc* replicates **(b)**, and Col and REF6-HA **(c)**.

(d) ChIP-qPCR validation of REF6-binding at the *BRox1* and *HB23* loci. The individual data points are shown as dots. Error bars indicate mean ± SE from three independent experiments. Source data are provided as a Source Data file.

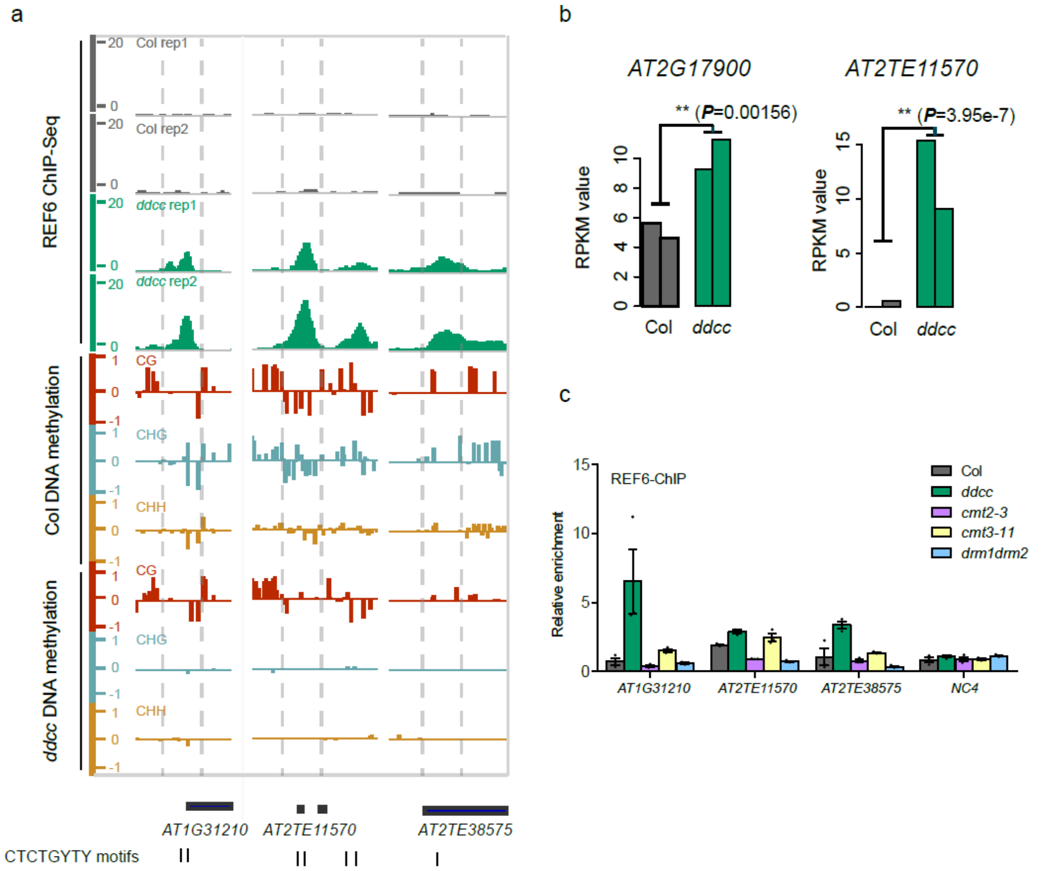

Supplementary Figure 6. DNA methylation represses REF6 binding at specific loci.
(a) Genome-browser view of ectopic REF6-binding sites in *ddcc* mutants.
(b) Barplot of the RPKM value for *AT2G17900* and *AT2TE11570* from two replicates in Col and *ddcc* show significantly up-regulated gene expression level in *ddcc*. RNAseq data is from the published data from (Stroud et al, NSMB, 2014). edgeR Fisher's exact test was used to calculate the P value. **, P < 0.01; ****, P < 0.0001.
(c) ChIP-qPCR validation of REF6-binding at *AT1G31210*, *AT2TE11570*, and *AT2TE38575* loci, using ChIP samples of another biological replicate, in wild-type Col and the *ddcc*, *cmt2-3*, *cmt3-11*, and *drm1 drm2* mutants. *NC4* was used as the negative control. ChIP-qPCR was performed in three technical replicates. Error bars indicate mean ± SE from three independent experiments. The individual data points are shown as dots. Source data are provided as a Source Data file.

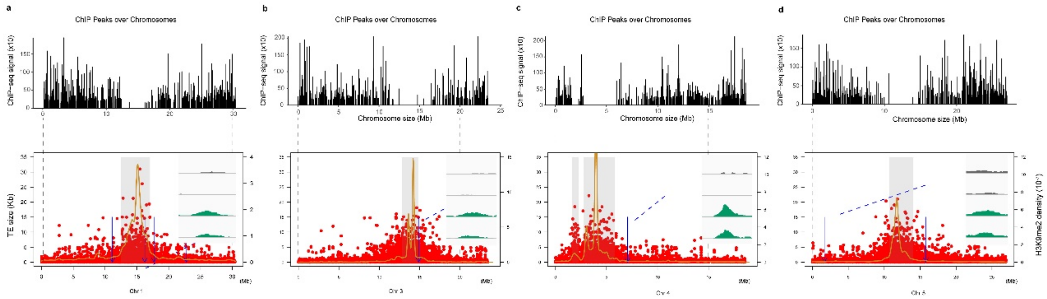

Supplementary Figure 7. REF6-binding sites in Col and the *ddcc* mutant across *Arabidopsis* chromosomes.

(a–d) Distribution of REF6 ChIP-seq signal, TEs (red dot) by size (kb), and H3K9me2 density (yellow line) across chromosomes 1, 2, 4 and 5. Blue lines with arrows indicate ectopic binding sites in the *ddcc* mutant, one of which is shown as an expanded view in the top right corner of each panel. Gray shading covering the area of high H3K9me2 density represent the heterochromatin regions.

Supplementary Table 1. Summary of ChIP-BS-seq and ChIP-seq data analysis.

Library	Library Type	Total reads	Clean reads	BS-seeker unique-hits reads (% of total reads)
ref6 ChBS-Input-rep 1	ChIP-BS	41,210,672	41,204,760	14,772,837 (35.85%)
Col REF6-ChBS-rep 1	ChIP-BS	39,368,136	39,359,562	10,366,454 (26.34%)
ddcc REF6-ChBS-rep1	ChIP-BS	38,909,862	38,892,547	11,471,341 (29.50%)
ref6 ChBS-Input-rep 2	ChIP-BS	42,465,152	42,453,991	17,662,835 (41.60%)
Col REF6-ChBS-rep 2	ChIP-BS	39,444,734	39,436,424	13,961,957 (35.40%)
ddcc REF6-ChBS-rep 2	ChIP-BS	38,161,444	38,155,586	9,783,749 (25.64%)
**Library**	**Library Type**	**Total reads**	**Total mapped reads (% of total)**	**Unique mapped reads (% of total mapped)**
ref6-REF6-IP-rep 1	ChIP-seq	5,718,280	2,490,406 (43.55%)	1,733,935 (69.62%)
Col REF6-IP-rep 1	ChIP-seq	3,754,404	2,251,278 (59.96%)	1,724,947 (76.62%)
ddcc REF6-IP-rep 1	ChIP-seq	5,199,673	2,855,213 (54.91%)	2,199,248 (77.02%)
ref6-REF6-IP-rep 2	ChIP-seq	22,145,136	9,986,503 (45.10%)	7,053,860 (70.63%)
Col REF6-IP-rep 2	ChIP-seq	22,904,799	11,978,451 (52.30%)	8,717,474 (72.78%)
ddcc REF6-IP-rep 2	ChIP-seq	22,686,457	12,127,221 (53.46%)	9,068,274 (74.78%)

Supplementary Table 2. Crystallographic data collection and refinement statistics.

	ZnF2-4-NAC004<br>ZnF2-4 (6JNL)<br>5′-TTCTCTGTTTTG-3′<br>3′-AAGAGACAAAAC-5′	ZnF2-4-NAC004_mC$_1$<br>ZnF2-4 (6JNN) [*]<br>5′-TTMTCTGTTTTG-3′<br>3′-AAGAGACAAAAC-5′	ZnF2-4-NAC004_mC$_3$<br>ZnF2-4 (6JNM)<br>5′-TTCTMTGTTTTG-3′<br>3′-AAGAGACAAAAC-5′
**Data collection**			
Space group	0.9785	0.9774	0.9774
Cell dimensions	$P4_32_12$	$P3_1$	$P4_3$
a, *b*, *c* (Å)	89.16, 89.16, 72.60	70.97,70.97,141.08	88.52, 88.52, 72.33
α, β, γ (°)	90, 90, 90	90, 90, 120	90, 90, 90
Resolution (Å)	30–2.05 (2.12–2.05) [a]	30–2.60 (2.69–2.60)	30–2.05 (2.12–2.05)
R_{merge}	0.053(0.59)	0.146(0.74)	0.103(1.02)
$I / \sigma I$	52.2(1.9)	13.7(2.1)	24.2(2.1)
Completeness (%)	99.9(99.9)	99.9(99.9)	100.0(100.0)
Redundancy	17.7(9.6)	6.7(5.2)	12.3(10.3)
**Refinement**			
Resolution (Å)	30.00–2.15	30.00–2.60	30.00–2.05
No. reflections	15643 (1131)	18614 (550)	31212 (963)
R_{work} / R_{free}	0.20/0.25	0.25/0.27	0.20/0.23
No. atoms	1259	4942	2654
Protein	739	2940	1478
Ligand/Water	34	12	174
DNA	486	1990	1002
B-factors	65.583	54.331	28.273
R.m.s. deviations			
Bond lengths (Å)	0.003	0.012	0.004
Bond angles (°)	1.468	1.976	1.499
**Ramachandran plot**			
Favored/allowed (%)	94.25/4.60	94.54/5.17	98.28/1.72

[a] Highest-resolution shell is shown in parentheses.

[*] M in the DNA sequence stands for 5-methylcytosine.

Supplementary Table 3. ITC fitting parameters.

DNA probe	DNA sequence	N	K	Kd（nM）	△H cal/mol	T△S cal/mol
NAC004	5'-ttctctgttttg-3'<br>3'-aagagacaaaac-5'	1.17±0.00423	1.36E7±1.42E6	73.5±7	-6145±34.82	300
*NAC004*_5mC1	5'-ttc(5mC)tctgttttg-3'<br>3'-aagagacaaaac-5'	1.06±0.00554	1.52E7±2.40E6	65.7±4.9	-2856±25.33	582.5
*NAC004*_5mC3	5'-ttctc(5mC)tgttttg-3'<br>3'-aagagacaaaac-5'	1.03±0.0132	9.06E6±2.64E6	110.3±3.8	-2162±43.59	615
*NAC004*_5mC1+5mC3	5'-ttc(5mC)tc(5mC)tgttttg-3'<br>3'-aagagacaaaac-5'	N.D.				
*NAC004*_5mC5	5'-ttctctgttttg-3'<br>3'-aagagac(5mC)aaaac-5'	N.D.				

Supplementary Table 4. Ectopic binding sites of REF6 in *ddcc* mutant.

Chr	Peak_start	Peak_end	Gene_ID	Type	Gene_annotation
Chr1	11,147,736	11,148,077	AT1TE36030	transposable_element	ATCOPIA25
Chr1	11,152,736	11,152,894	AT1TE36040	transposable_element	ATCOPIA52
Chr1	16,238,193	16,238,503	AT1G43145	protein_coding	unkown protein
Chr1	22,795,991	22,796,210	AT1G61732	microRNA	encodes a microRNA
Chr1	17,680,960	17,681,313	AT1TE58705	transposable_element	LTR/copia
Chr2	1,468,879	1,469,430	NA	intergenic	
Chr2	2,578,356	2,578,725	AT2TE11570	transposable_element	ATCOPIA50
Chr2	2,580,213	2,580,887	AT2G06500	protein_coding	hAT family dimerisation domain
Chr2	9,122,126	9,122,682	AT2TE38575	transposable_element	ATCOPIA74
Chr2	7,776,685	7,777,792	AT2TE32120	transposable_element	LINE/L1
Chr3	14,861,102	14,861,453	AT3TE60635	transposable_element	ATCOPIA34
Chr4	7,060,763	7,061,163	AT4TE30625	transposable_element	ATLINEIII
Chr5	1,683,301	1,683,690	AT5G05630	protein_coding	Polyamine uptake transporter 3
Chr5	15,813,699	15,814,483	AT5TE57090	transposable_element	ATHATN2

Supplementary Table 5. Oligonucleotides used in this study.

Gene	Primer sequences (5' to 3')
NAC04(AT1G02230)	GGGAGAATCTCAAACTTTCTCTGTTTTGTCTTTAGAAA CTTTGAAATATC
AT4TE30625	CTATCAAATCTTCTATCACCACTCTGTTTTATGGCCATC AAATGATTTCG
AT2TE32120-F	TCATCTGAGCGGAGCTTCTACC
AT2TE32120-R	CAGAGGTTGATTATCTTCAAGAGACATG
AT5TE57090-F	GGATCGAAATAGAAGAGAAAGTCCAG
AT5TE57090-R	GACGCAGCTTCTTCTCTCGACT
AT2TE11570-F	GCAAGACTGAAACAGAGAGAATAAGAAG
AT2TE11570-R	CTCTTCCATGTTGTTTAGTGGATTCC
AT1G31210-F	GCAAAACGCCCCTCCTCTGTAC
AT1G31210-R	CGACATACACCCTAGTTACATTGTTTG
AT2TE38575-R	GCTCCTCTCTCTTCTTCCGTACTCTG
AT2TE38575-F	GTAACCTTGCCTATACGGCTATACATG
HB23-F	GCCATCCACCGACAACTACAAC
HB23-R	ACTGTTCTCTTCTTTCACCATTCTCTG
NC4-F	TGGCAGGTGTCAATATCAAGC
NC4-R	AATAAGGCAGCGTTTGGAGTG
BR6ox1-F	GCCATCTCTCTCTCCCTCTCACTC
BR6ox1-R	CATCACCATCATTGCTCCCATCTC
NAC004P-F	TTCTCTGTTTTG
NAC004P-R	CAAAACAGAGAA
NAC004P-5mC$_1$	TT**5mC**TCTGTTTTG
NAC004P-5mC$_3$	TTCT**5mC**TGTTTTG
NAC004P-5mC$_5$	CAAAA**5mC**AGAGAA
NAC004P-5mC$_1$+5mC$_3$	TT**5mC**T**5mC**TGTTTTG
