# Supplementary material for: DNA methylation repels targeting of Arabidopsis REF6
Source: Nat Commun. 2019 May 2;10:2063. doi: 10.1038/s41467-019-10026-1 (PMC6497721; doi:10.1038/s41467-019-10026-1)
Supplement: Supplementary file 3 — Reporting Summary [file 41467_2019_10026_MOESM3_ESM.pdf]

nature research

Corresponding author(s): Xiaofeng Cao and Jinbiao Ma

Last updated by author(s): Jan 28, 2019

Reporting Summary

Nature Research wishes to improve the reproducibility of the work that we publish. This form provides structure for consistency and transparency in reporting. For further information on Nature Research policies, see Authors & Referees and the Editorial Policy Checklist.

Statistics

For all statistical analyses, confirm that the following items are present in the figure legend, table legend, main text, or Methods section.

n/a	Confirmed	
☐	☒	The exact sample size (*n*) for each experimental group/condition, given as a discrete number and unit of measurement
☐	☒	A statement on whether measurements were taken from distinct samples or whether the same sample was measured repeatedly
☐	☒	The statistical test(s) used AND whether they are one- or two-sided<br>*Only common tests should be described solely by name; describe more complex techniques in the Methods section.*
☒	☐	A description of all covariates tested
☐	☒	A description of any assumptions or corrections, such as tests of normality and adjustment for multiple comparisons
☐	☒	A full description of the statistical parameters including central tendency (e.g. means) or other basic estimates (e.g. regression coefficient) AND variation (e.g. standard deviation) or associated estimates of uncertainty (e.g. confidence intervals)
☐	☒	For null hypothesis testing, the test statistic (e.g. *F*, *t*, *r*) with confidence intervals, effect sizes, degrees of freedom and *P* value noted<br>*Give P values as exact values whenever suitable.*
☒	☐	For Bayesian analysis, information on the choice of priors and Markov chain Monte Carlo settings
☒	☐	For hierarchical and complex designs, identification of the appropriate level for tests and full reporting of outcomes
☐	☒	Estimates of effect sizes (e.g. Cohen's *d*, Pearson's *r*), indicating how they were calculated

Our web collection on statistics for biologists contains articles on many of the points above.

Software and code

Policy information about availability of computer code

Data collection	Data of BS-seq in different mutants were download from GEO (GSE39901 and GSE51304). X-ray data collection and processing. HKL3000.
Data analysis	PHENIX; Phaser programs; Coot; model building; PyMOL. Paired-end sequencing reads from ChIP-seq were mapped to the Arabidopsis thaliana TAIR10 reference genome using Bowtie2 (version 2.2.8) . ChIP-seq and ChIP-BS-seq peaks were called using MACS2 (v 2.1.1). Peaks binding signal and methylation level from ChIP-BS-Seq were processed by deepTools2. Differentially methylated regions (DMRs) were identified using the R package DMRcaller.

For manuscripts utilizing custom algorithms or software that are central to the research but not yet described in published literature, software must be made available to editors/reviewers. We strongly encourage code deposition in a community repository (e.g. GitHub). See the Nature Research guidelines for submitting code & software for further information.

Data

Policy information about availability of data

All manuscripts must include a data availability statement. This statement should provide the following information, where applicable:

- Accession codes, unique identifiers, or web links for publicly available datasets
- A list of figures that have associated raw data
- A description of any restrictions on data availability

X-ray structures (coordinates and structure factor files) of REF6 ZnFs with bound DNA have been submitted to PDB under accession numbers 6JNL, 6JNN, 6JNM represents for ZnF2-4-NAC004, ZnF2-4-NAC004_5mC1, ZnF2-4-NAC00_5mC3, respectively. ChIP-seq data sets generated in this study have been deposited in the Gene Expression Omnibus (GEO) under accession GSE111830.

Field-specific reporting

Please select the one below that is the best fit for your research. If you are not sure, read the appropriate sections before making your selection.

☒ Life sciences ☐ Behavioural & social sciences ☐ Ecological, evolutionary & environmental sciences

For a reference copy of the document with all sections, see nature.com/documents/nr-reporting-summary-flat.pdf

Life sciences study design

All studies must disclose on these points even when the disclosure is negative.

Sample size	DNA methylation and REF6-binding were measured with bulk-samples. For ChIP-BS-seq and ChIP-seq, about 3 g of seedlings from about 10 plates were collected as one sample, and two independent collections were performed for two independent biological replicates, respectively.
Data exclusions	No data was excluded.
Replication	ChIP-seq and ChIP-BS-seq experiments were performed with two independent replicates, and the two biological replicates were highly reproducible. For ITC and real-time PCR experiments, two independent replicates were performed with reproducible results.
Randomization	Randomization was not required.
Blinding	The investigators were not blinded during data collections and analysis.

Reporting for specific materials, systems and methods

We require information from authors about some types of materials, experimental systems and methods used in many studies. Here, indicate whether each material, system or method listed is relevant to your study. If you are not sure if a list item applies to your research, read the appropriate section before selecting a response.

Materials & experimental systems

n/a	Involved in the study	
☐	☒	Antibodies
☒	☐	Eukaryotic cell lines
☒	☐	Palaeontology
☒	☐	Animals and other organisms
☒	☐	Human research participants
☒	☐	Clinical data

Methods

n/a	Involved in the study	
☐	☒	ChIP-seq
☒	☐	Flow cytometry
☒	☐	MRI-based neuroimaging

Antibodies

Antibodies used	anti-REF6 monoclonal antibody (custom antibody from Abmart, Shanghai, China) was used for ChIP-seq and ChIP-BS-seq.
Validation	ChIP-seq results using REF6-antibody were highly correlated (r=0.83) with those REF6-HA ChIP-seq using anti-HA antibody. REF6 target genes were efficiently enriched in Col in comparison with ref6 mutant, indicating that the anti-REF6 antibody worked well.

ChIP-seq

Data deposition

☒ Confirm that both raw and final processed data have been deposited in a public database such as GEO.

☒ Confirm that you have deposited or provided access to graph files (e.g. BED files) for the called peaks.

Data access links<br>*May remain private before publication.*	https://www.ncbi.nlm.nih.gov/geo/query/acc.cgi?acc=GSE111830
Files in database submission	Col_REF6_ChIPSeq_rep1: anti-REF6 ChIP-seq replicate1 in wild type; ddcc_REF6_ChIPSeq_rep1: anti-REF6 ChIP-seq replicate 1 in ddcc mutant; ref6_REF6_ChIPseq_rep1: anti-REF6 ChIP-seq replicate1 in wild ref6-5 mutant; Col_REF6_ChIPSeq_rep2: anti-REF6 ChIP-seq replicate2 in wild type; ddcc_REF6_ChIPSeq_rep2: anti-REF6 ChIP-seq replicate 2 in ddcc mutant; ref6_REF6_ChIPseq_rep2: anti-REF6 ChIP-seq replicate2 in wild ref6-5 mutant; WT REF6-ChBS-rep1: bisulfite sequencing replicate1 after anti-REF6 ChIP enrichment in wild type; ddcc REF6-ChBS-rep1: bisulfite sequencing replicate1 after anti-REF6 ChIP enrichment in ddcc mutant; ref6 ChBS-Input1-rep1: bisulfite sequencing replicate1 after anti-REF6 ChIP enrichment in ref6-5 mutant; WT REF6-ChBS-rep2: bisulfite sequencing replicate2 after anti-REF6 ChIP enrichment in wild

	type; ddcc REF6-ChBS-rep2: bisulfite sequencing replicate2 after anti-REF6 ChIP enrichment in ddcc mutant; ref6 ChBS-Input1-rep2: bisulfite sequencing replicate2 after anti-REF6 ChIP enrichment in ref6-5 mutant;
Genome browser session (e.g. UCSC)	N/A

Methodology

Replicates	ChIP-seq and ChIP-BS-seq experiments were performed with two independent replicates.
Sequencing depth	ChIP-seq and ChIP-BS-seq were all sequenced in paired-end. Individually, Col_REF6_ChIPSeq_rep1 had 3754404 total reads with 1724947 unique mapped reads; ddcc_REF6_ChIPSeq_rep1 had 5199673 total reads with 2199248 unique mapped reads; ref6_REF6_ChIPseq_rep1 had 5718280 total reads with 1733935 unique mapped reads; Col_REF6_ChIPSeq_rep2 had 22904799 total reads with 8717474 unique mapped reads; ddcc_REF6_ChIPSeq_rep2 had 22686457 total reads with 9068274 unique mapped reads; ref6_REF6_ChIPseq_rep2 had 22145136 total reads with 7053860 unique mapped reads; WT REF6-ChBS-rep1 had 41210672 total reads with 14772837 unique mapped reads; ddcc REF6-ChBS-rep1 had 39368136 total reads with 10366454 unique mapped reads; ref6 ChBS-Input1-rep1 had 38909862 total reads with 11471341 unique mapped reads;WT REF6-ChBS-rep2 had 42465152 total reads with 17662835 unique mapped reads; ddcc REF6-ChBS-rep2 had 39444734 total reads with 13961957 unique mapped reads; ref6 ChBS-Input1-rep2 had 38161444 total reads with 9783749 unique mapped reads.
Antibodies	anti-REF6 monoclonal antibody (custom antibody from Abmart, Shanghai, China) was used for ChIP-seq and ChIP-BS-seq.
Peak calling parameters	ChIP-seq and ChIP-BS-seq peaks were called using MACS2 with the "--gsize 119000000 --keep-dup 1" options.
Data quality	FastQC (v0.11.5) was applied to ensure data quality for checking adapter sequences, low quality bases. samtools (v0.1.8) was used to remove duplicated reads. Qualified peaks were filtered by MACS2 q-value (q-value <= 0.05).
Software	Bowtie2 (v2.2.8); BEDTools (v2.17.0); MACS2 (v2.1.1); IGV genome browser (v2.3.72); MAnorm, ChIP-seq differentially binding analysis; deepTools2(v3.0.2); R (v3.2.5); BS-Seeker2; samtools (v0.1.8); ChIPseeker for peak annotation (Bioconductor package); GeneOverlap (Bioconductor package).
